# Location of ischemia and ischemic pain intensity affect spatiotemporal parameters and leg muscles activity during walking in patients with intermittent claudication

**DOI:** 10.1038/s41598-021-86351-7

**Published:** 2021-03-24

**Authors:** Céline Guilleron, Pierre Abraham, Bruno Beaune, Camille Pouliquen, Samir Henni, Sylvain Durand

**Affiliations:** 1 Le Mans Université, Movement—Interactions—Performance, MIP, EA 4334, 72000 Le Mans, France; 2 Sports Medicine, University Hospital of Angers, 4 rue Larrey, 49100 Angers, France; 3 UMR CNRS 6015 INSERM 1083, University of Angers, 4 rue Larrey, 49100 Angers, France; 4 Department of Vascular Medicine, University Hospital of Angers, 4 rue Larrey, 49100 Angers, France; 5 Laboratory “Movement, Interactions, Performance”, MIP, EA 4334, Le Mans University, Avenue Olivier Messiaen, 72085 Le Mans Cedex 9, France

**Keywords:** Atherosclerosis, Peripheral vascular disease

## Abstract

The ways in which locations of ischemia and ischemic pain affect spatiotemporal gait parameters and leg electromyographic activity during walking have never been investigated in patients with peripheral arterial disease presenting intermittent claudication. Two groups were classified according to unilateral location of ischemia (distal, n = 10, or proximo-distal, n = 12). Patients described pain and three gait phases—initial pain-free, onset of pain and maximum pain—were analyzed. Patients with proximo-distal ischemia walked less (230 ± 111 m vs 384 ± 220 m), with increased step length, step time (+ 5.4% and + 5.8%) and reduced cadence (− 8.2%), than patients with distal ischemia. In both, the peaks of vertical ground reaction force were reduced in maximum pain (Peak1-distal: − 11.4%, Peak1-proximo-distal: − 10.3%; Peak2-distal: − 11.8%, Peak2-proximo-distal: − 9.0%). In the proximo-distal group, tibialis anterior activation peak and time were lower than in the distal group (− 4.5% and − 19.7%). During the maximum pain phase, this peak decreased only in the proximo-distal group (− 13.0%), and gastrocnemius medialis activation peak and time decreased in both groups (− 2.5% in distal and − 4.5% in proximo-distal). Thus, proximo-distal ischemia leads to more adverse consequences in gait than distal ischemia only. Increasing ischemic pain until maximum, but not onset of pain, induced gait adaptations.

## Introduction

Peripheral arterial disease (PAD) is a cardiovascular condition caused by atherosclerosis. It affected more than 202 million people in the world in 2010 with an 8 to 12% prevalence after 60 years of age^1^. The most common symptom of lower-limb PAD is intermittent claudication (IC) which manifests as leg muscle ischemic pain progressively occurring during walking. It can cause the patient to stop activity, until pain relief. Pain in IC is based on temporary ischemia due to arterial stenosis or thrombosis, limiting local blood flow^2^. Ischemia can appear in different locations. Ischemia is classified as “distal” when localized in the calf or “proximo-distal” when localized both in the calf and the thigh and/or buttock. Consequently, depending on the symptom intensity, patients suffering from IC have reduced lower limb mobility, maximum walking distance (distance patients can travel until they have to stop due to ischemic pain) and altered spatiotemporal gait parameters^3–5^. Previous studies showed that IC patients have a diminished plantar propulsion force during walking with the prolongation of claudication pain^6,7^, compared to a control group. Conversely, one study found no difference between pain-free and painful phases^8^. Therefore, the relationships between ischemia, claudication and pain development are not fully understood.

PAD causes not only muscular but also neuromuscular damage^9^. Decreased muscle strength, partial denervation of legs muscles and loss in nerve conduction velocity have been described^9^. Despite this, contradictory results exist concerning the impact of IC on *gastrocnemius medialis* (GM) activation. One study reported no change in the activation capacity during maximum voluntary contractions^10^ and one study reported an increase in electromyography amplitudes during walking^11^. To our knowledge, only one study has described muscle activation modalities in IC patients during walking by using surface electromyography (sEMG)^11^. These authors showed that *tibialis anterior* (TA) muscle activity duration decreased during IC, and sEMG amplitudes increased for TA and GM muscles.

Therefore, the aim of this study was to characterize gait pattern and muscle activation modalities in the ischemic leg of IC patients presenting unilateral lower limb distal (DIS) or proximo-distal (PROXDIS) ischemia during the pain-free, onset of pain and maximum pain periods of a walking-treadmill test. This characterization will help to adapt and optimize rehabilitative care according to ischemia location.

Our main hypothesis is that the location of ischemia affects leg muscles and influences the aforementioned parameters differently. Specifically, PROXDIS ischemia is more susceptible to affect the gait pattern than DIS ischemia. Furthermore, despite previous investigations describing the impact of ischemic pain on spatiotemporal gait parameters or neuromuscular activation in IC patients, little is known about the effect of ischemic pain intensity on these parameters. We believe that these alterations appear immediately at the onset of pain and increase with ischemic pain intensity until its maximum.

## Methods

### Participants

**Figure 1:**
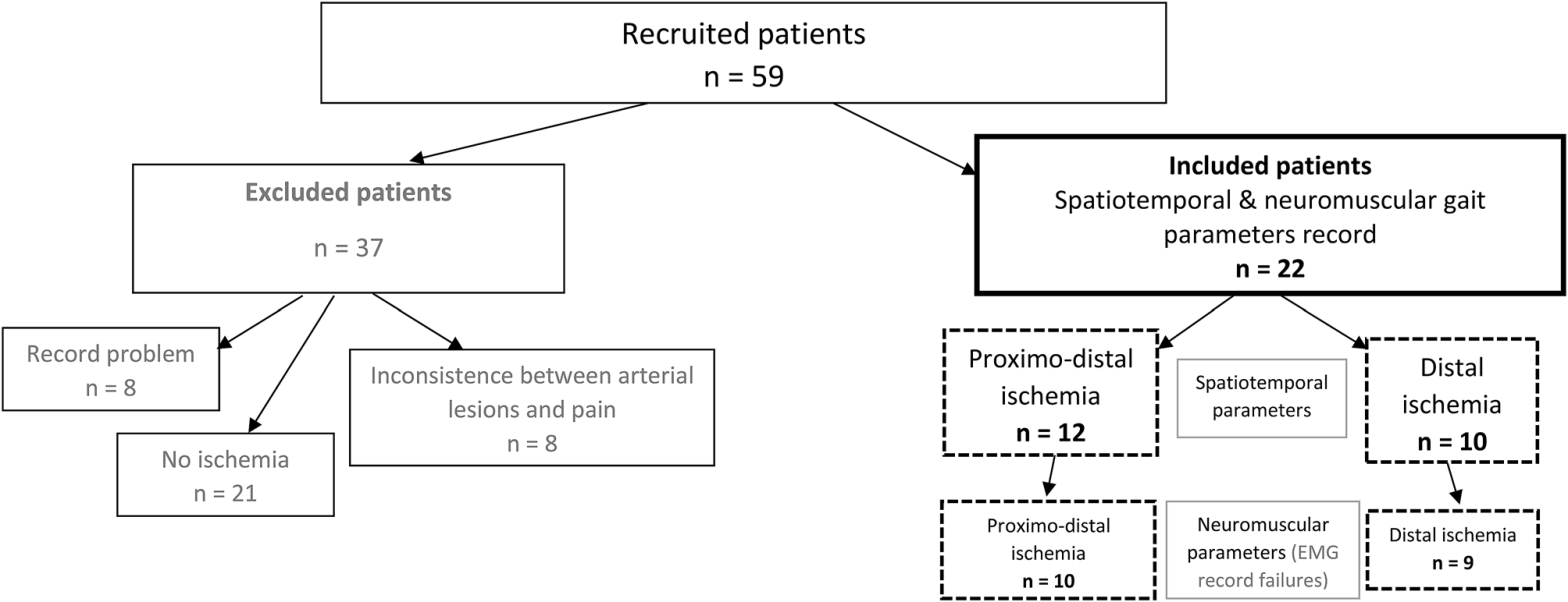
Flowchart of the population studied.

Symptomatic IC patients diagnosed with PAD (Ankle Brachial Index: ABI ≤ 0.90), and exhibiting claudication, were recruited during an examination from the functional vascular investigations service in Angers university hospital (France). All 59 patients underwent Doppler and/or angiography and/or computed tomography examination (Fig. 1). We recorded: age (years), height (m), weight (kg), body mass index (kg/m^2^), ABI of the ischemic leg, smoking status, presence of treated hypertension (number (n)), diabetes (n), hypercholesterolemia (n), distance to onset of pain (m) and maximum walking distance (m). The functional investigation included a treadmill-walking test to confirm the presence and location of ischemia using a transcutaneous oxygen pressure recording with an index indicating ischemia (delta from rest oxygen pressure (DROP) < − 15 mmHg) as previously described^32^. Patients were included if the test confirmed that they suffered unilateral (one leg) distal or proximo-distal ischemia (DIS and PROXDIS patients), and 22 male patients were finally included (Fig. 1). Concordance between patients’ description of pain (due to ischemia) during walking and location of ischemia was checked. Patients were not included if they presented a comorbid condition that might influence gait pattern, such as previous vascular surgery on lower extremities (bypass, percutaneous angioplasty), lower extremity amputation, severe osteoarthritis, knee or hip prosthesis, severe cardiopulmonary problems (i.e., chronic obstructive pulmonary disease), neurological diseases (i.e., diabetes neuropathy), Parkinson’s disease, stroke, or use of walking aids. Patients were excluded if unable to walk ten meters in less than 13 s and if they had pain when resting. After the testing protocol, other patients were also excluded from analysis if they did not present ischemia during the test, or if they showed discordance between arterial lesion location and pain location description (Fig. 1). Participants eligible for inclusion were informed and gave their written consent. All experimental protocols were approved by the local ethics committee (Committee for Protection of Persons Ouest II). The protocol was promoted by the Angers university hospital and registered in Clinicaltrials.gov website under reference: NCT02754804, April 28th, 2016 (https://clinicaltrials.gov/ct2/show/NCT02754804).

### Study protocol

#### Walking test

The treadmill-walking test was a constant load procedure^39^. The speed reached 3.2 km/h (i.e., 2 miles/h) in one minute, with a 10% slope. Speed was then stabilized at 3.2 km/h. Patients were instructed to describe pain development in lower limbs (location and intensity) during walking. They were encouraged to perform as long as possible to reproduce claudication symptoms. Exercise was stopped at the patient’s request (pain or other difficulties to maintain the effort) or after 15 min. During this test, we checked the concordance between the description of the pain and DROP values (obtained by recording the transcutaneous oxygen pressure, carried out during the walking test).

#### Gait parameters

Gait parameters in the ischemic leg were recorded at 200 Hz with an instrumented treadmill (Zebris FDM-T, Germany, 2011). This gait analysis system has a recording surface of 108 × 47 cm with 7168 barometric sensors each approximately 0.85 cm^2^. We recorded the following parameters:

- Temporal parameters: step time (s), cadence (step/min), single leg stance and total stance phase in % of the gait cycle,
- Spatial parameters: step length (m), step width (m), foot progression angle (FPA), defined as the angle (degree) between the longitudinal axis of the foot and the line of progression during walking,
- Dynamometric parameters: peaks of vertical ground reaction forces (N): first peak corresponding to heel contact and second peak corresponding to toe-off during a gait cycle (corresponding to braking and propulsive forces, respectively).

Gait parameters were analyzed on ten successive gait cycles at three different phases in function of pain intensity:

- The pain-free phase was the first ten gait cycles at the beginning of the walking test, before the onset of pain.
- The onset of pain was the first ten gait cycles after the patient reported pain arising during the walking test
- The maximum pain was the last ten gait cycles before the patient had to stop the walking test because of the pain.

To limit anthropometry-based differences, we normalized step length by the height (m) of the patients and peak forces by body weight (N).

#### Electromyography

sEMG is a non-invasive technique to assess muscle activity during walking. TA and GM muscle activity were investigated in the ischemic leg (Zebris EMG measuring system, Germany, 2011). These muscles were selected because: (1) they are highly involved in ankle movement during walking, (2) calf (and consequently GM) is the site most often reported by patients when describing pain location during claudication and (3) TA is the GM antagonist muscle. Before placement of the sEMG probes, skin was shaved and cleaned with alcohol. The probes were placed according to “Surface EMG for Non-Invasive Assessment of Muscles” recommendations^40^. sEMG signals were recorded at 1000 Hz and stored by the Zebris software (Zebris EMG measuring system, Germany, 2011, version 1.16).

sEMG signals were band-pass filtered between 15 and 499 Hz with a second-order Butterworth filter and full wave, rectified to create linear sEMG envelopes. sEMG data were normalized to a 100% gait cycle using gait parameters synchronized with sEMG signals. To determine muscle activation level, the Root Mean Square (RMS) was calculated and normalized with respect to the RMS peak values of 10 gait cycles during the pain-free phase, according to the *“peak dynamic method”*^41^. The sEMG process was made with a custom script in Matlab 2016a (MathWorks Inc, Natick, MA, USA). An average sEMG profile was obtained from 10 gait cycles for each patient at the phases of the gait studied (beginning and end of individual gait cycle were identified by heel contact). A threshold for muscle onset, fixed as recommended at 20% of the peak, was used to detect the time of activation of each muscle (% of gait cycle)^41^.

### Statistical analysis

Data are expressed as mean(SD (standard deviation)). All the statistical procedures were performed using Statistica software V_10.0_ (StatSoft, Inc. 2011. STATISTICA—data analysis software system—version 10). Levene’s test and Fisher’s post-hoc least significant difference (PLSD) analysis were performed to assess variance homogeneity of the series. Prevalence between groups was compared with a Chi-squared test.

Differences in anthropometric measurements (ABI, Body Mass Index), gait parameters and EMG signals were studied according to the locations of ischemia (PROXDIS vs DIS), the intensity of pain (pain-free, onset of pain and maximum pain) and between legs, using a three-way ANOVA procedure; α power was indicated for each test. When normality of the distribution was not assessed, a Kruskal–Wallis test was done. For all tests a significant difference was fixed at P < 0.05.

### Ethical approval

All procedures performed in studies involving human participants were in accordance with the ethical standards of the institutional and/or national research committee (CPP Ouest II) and with the 1964 Helsinki declaration and its later amendments or comparable ethical standards.

### Informed consent

The procedure, purpose, and risks associated with this study were explained to the subjects, and written informed consent was obtained.

## Results

### Participants

A total of 59 PAD patients with IC were recruited. After the treadmill-walking test, 37 were excluded because of inconsistency with our selection criteria (Fig. 1). Finally, 22 symptomatic IC patients diagnosed with PAD and unilateral claudication were included in the study. Ten presented a DIS ischemia (femoro-popliteal lesions) and 12 presented a PROXDIS ischemia (one patient had isolated common iliac lesions, all others had iliac and femoro-popliteal lesions). The baseline characteristics for IC patients are presented in Table 1. There was no difference concerning anthropometric characteristics between groups.

**Table 1:** Characteristics of the groups relative to locations of ischemia (*mean(SD)*).

Group/Characteristic	Distal (n = 10)	Proximo-distal (n = 12)
Age (years)	70 (8)	65 (5)
Height (m)	1.67 (0.07)	1.71 (0.10)
Mass (kg)	74 (18)	73 (14)
Body mass index (kg/m^2^)	26.1 (5.3)	24.7 (3.5)
Ankle brachial index	0.57 (0.16)	0.67 (0.17)
Proximal DROP (mmHg)	− 9 (5)	− 33 (12) *
Distal DROP (mmHg)	− 31 (11)	− 30 (11)
Smoking (n)	6	12
*Active smoker*	*4*	*8*
Hypertension (n)	4	2
Diabetes (n)	3	3
Hypercholesterolemia (n)	4	3
Pharmacological treatments (n)	10	12
*Antiaggregant*	8	9
*Antihypertensive*	9	11
*Lipid-lowering*	6	9
*Antidiabetic*	2	2
Distance for onset of pain (m)	140 (76)	84 (61)
Maximum Walking Distance (m)	384 (220)	230 (111)*

*P < 0.05 between distal and proximo-distal ischemia.

The average distance from which all patients experienced ischemic pain in the lower limbs was 109 ± 75 m. The average maximum claudication distance forcing the patients to stop walking was 306 ± 185 m and the patients with DIS ischemia walked significantly longer than those with PROXDIS ischemia (Table 1), although no difference in the ankle brachial index (ABI) was found between groups.

For both gait and EMG parameters, no differences were found between ischemic and healthy legs (not report).

### Gait parameters

There was no interaction effect in spatiotemporal gait parameters between location of ischemia and pain effects (Table 2).

**Table 2:** Gait parameters during the different phases of the gait in the ischemic leg, relative to locations of ischemia *(mean (SD))*.

Pain intensity	Pain-free			Onset of pain		Maximum pain			p-location	p-pain	p- pain-location	α location	α pain	α pain-location
Location/parameters	Distal	Proximo-Distal		Distal	Proximo-Distal	Distal	Proximo-Distal							
Step length (% height)	29.4 (4.4)	32.1 (3.2		30.3 (3.8)	33.0 (3.5)	32.1 (4.5)	33.6 (3.0)		**0.006**	0.520	0.903	**0.83**	0.15	0.10
Step time (s)	0.57 (0.07)	0.61 (0.08)		0.58 (0.08)	0.63 (0.07)	0.60 (0.09)	0.64 (0.08)		**0.004**	0.542	0.906	**0.84**	0.15	0.06
Step width (cm)	9.9 (2.9)	8.5 (2.2)		9.7 (2.1)	8.8 (2.2)	9.6 (2.5)	9.1 (1.8)		0.104	0.950	0.820	0.37	0.06	0.08
Cadence (step/min)	108.2 (15.0)	99.6 (12.8)		106.2 (13.5)	97.2 (10.4)	103.7 (13.5)	97.2 (11.7)		**0.010**	0.632	0.953	**0.75**	0.12	0.06
Foot progression angle (°)	10.6 (6.8)	8.1 (4.2)		11.4 (7.5)	8.8 (4.1)	13.4 (7.0)	10.4 (4.9)		**0.032**	0.404	0.980	**0.58**	0.20	0.05
Single leg stance (% GC)	35.3 (1.2)	35.3 (1.0)		35.6 (1.2)	35.4 (1.3)	34.9 (1.3)	35.1 (1.3)		0.950	0.535	0.940	0.05	0.15	0.06
Stance (% GC)	64.4 (2.0)	65.0 (0.9)		64.5 (2.1)	64.6 (1.3)	64.0 (2.1)	63.8 (1.2)		0.777	0.218	0.853	0.06	0.32	0.07
Peak Force 1 (% BW)	89.3 (8.5)	87.2 (7.9)		83.8 (7.3)	83.1 (7.5)	79.1 (8.8)	78.2 (7.2)		0.738	**0.003**	0.854	0.06	**0.89**	0.07
Peak Force 2 (% BW)	86.1 (9.0)	85.8 (7.2)		81.1 (6.6)	80.8 (7.5)	75.9 (7.6)	78.1 (5.1)		0.642	**0.001**	0.840	0.07	**0.96**	0.08

p-location: p value with three-way ANOVA for location of ischemia differences during all phases.

p-pain: p value with three-way ANOVA for differences between pain-free and maximum pain for both locations of ischemia.

p-interaction pain-location: p value for interaction effects between pain and location of ischemia.

*GC* gait cycle and *BW* body weight.

#### Influence of location of ischemia

Step length and step time were significantly higher (+ 5.4% and + 5.8% respectively; P < 0.05) in the PROXDIS group than in the DIS group, coinciding with a − 8.2% lower cadence at the fixed speed (Table 2). In addition, the foot progression angle (FPA) was significantly higher in the DIS group.

#### Impact of ischemic pain intensity

The onset of pain had no impact on gait parameters. Significant differences were found only between the pain-free and maximum pain phases for peak force 1 and 2 in both groups (lower braking and propulsive forces) (Table 2).

### Electromyography

Because sEMG records presented some failures for three patients, sEMG data analysis was performed in nine patients with DIS ischemia and ten with PROXDIS ischemia, only. Between location of ischemia and pain effects, there was no interaction effect for electromyographic parameters (Table 3).

**Table 3:** EMG parameters during the different phases of the gait in the ischemic leg, relative to locations of ischemia *(mean (SD))*.

Pain intensity	Pain-free		Onset of pain		Maximum pain		p-location	p-pain	p- pain-location	α location	α pain	α pain-location
Location of ischemia/EMG parameters	Distal	Proximo-distal	Distal	Proximo-distal	Distal	Proximo-distal						
TA activation peak	0.90(0.11)	0.93(0.14)	0.88(0.19)	0.87(0.16)	0.93(0.34)	0.79(0.16)	**0.016**	0.587	/	/	/	/
GM activation peak	0.94(0.08)	0.94(0.13)	0.96(0.15)	0.95(0.29)	0.92(0.21)	0.89(0.28)	0.691	**0.046**	/	/	/	/
TA activation time (% of GC)	45.6(8.1)	38.2(5.7)	45.6(10.0)	36.7(5.1)	46.6(9.0)	40.1(5.6)	**0.003**	0.876	0.971	**0.85**	0.07	0.05
GM activation time (% of GC)	34.5(8.1)	34.2(5.8)	32.9(8.7)	31.5(6.1)	30.4(8.6)	29.7(4.9)	0.521	**0.049**	0.956	0.10	**0.58**	0.06

p-location: p value with three-way ANOVA for location differences during all phases.

p-pain: p value with three-way ANOVA for differences between pain-free and maximum pain for both locations of ischemia.

p-interaction pain-location: p value for interaction effects between pain and location of ischemia.

*GC* Gait Cycle./: Kruskal Wallis test was applied.

#### Influence of location of ischemia

TA showed significant differences between locations for the activation peak (DIS activation peak was 4.5% higher than PROXDIS) and the activation time (DIS activation time was 19.7% higher than PROXDIS) (Table 3).

#### Impact of ischemic pain intensity

**Figure 2:**
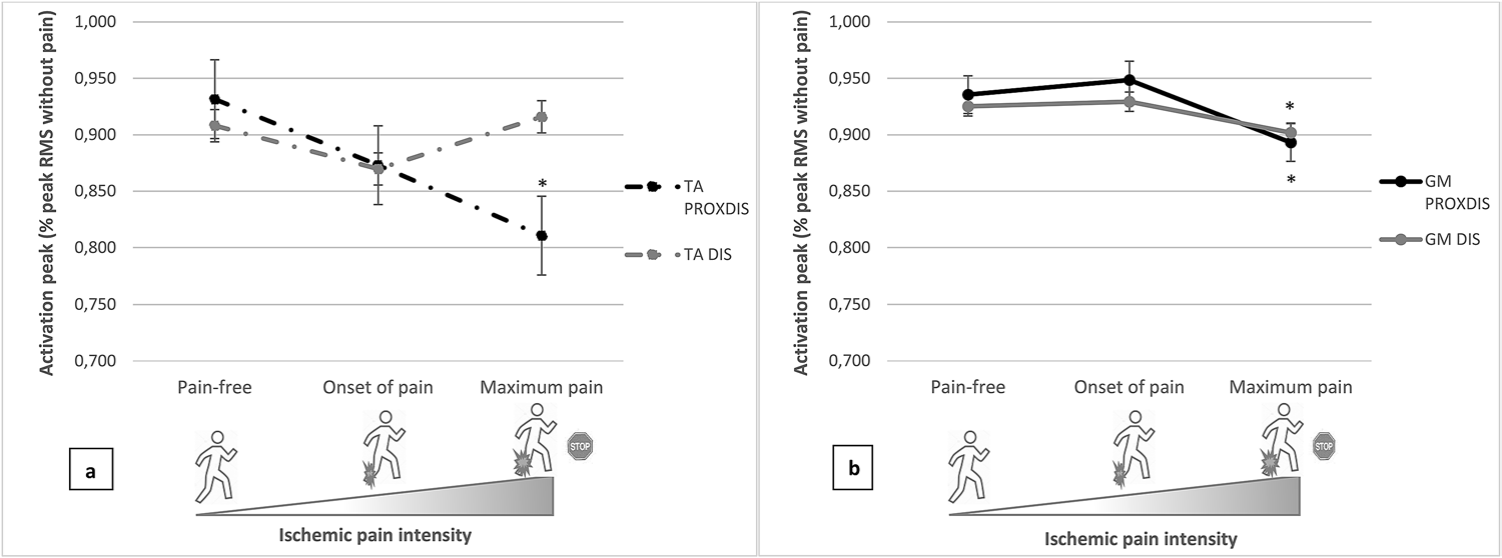
Activation peak of *tibialis anterior* (TA) (**a**) and *gastrocnemius medialis* (GM) (**b**) according to ischemic pain intensity and location of ischemia as a percentage of the peak Root Mean Square (RMS) during the Pain-free phase in the ischemic leg. Values are mean and standard deviation. *P < 0.05 between “Pain-free” and “Maximum pain” phases with a Kruskall Wallis test.

**Figure 3:**
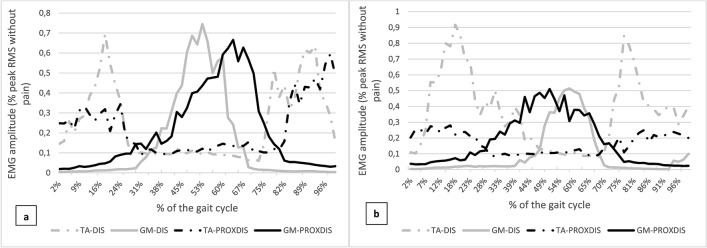
Electromyographic (EMG) amplitude (as a percentage of the peak Root Mean Square (RMS) during the Pain-free phase) in the ischemic leg of *tibialis anterior* (TA) and *gastrocnemius medialis* (GM) during the pain-free phase (**a**) and maximum pain phase (**b**) during a gait cycle, in a patient with distal ischemia (DIS) and a patient with proximo-distal ischemia (PROXDIS).

Onset of pain did not induce any change in sEMG parameters compared to the pain-free phase. Between the pain-free and the maximum pain phases, three sEMG parameters were affected (Table 3): the TA activation peak in the PROXDIS group only (decreasing by 13.0%, Fig. 2, Table 3, P = 0,035), the GM activation peak (decreasing by 2.5% in DIS and 4.5% in PROXDIS, Fig. 2, Table 3) and the GM activation time in both groups (decreasing by 8.6% in DIS and 14.5% in PROXDIS, Table 3). Examples of patient data are shown in Fig. 3.

## Discussion

To our knowledge, this study is the first to have characterized the influence of ischemia location and ischemic pain intensity on gait parameters and neuromuscular activation in patients presenting IC during walking.

Our main finding is that a PROXDIS ischemia location in the lower limbs induces greater gait alterations than DIS ischemia. In addition, ischemic pain affects gait parameters and neuromuscular activation with pain prolongation (and thus increased pain intensity) until maximum pain but not at the onset of pain.

Previous studies have investigated the impact of IC on gait parameters^12,13^, but none have differentiated between patients suffering from PROXDIS and DIS ischemia. We show that when patients have PROXDIS ischemia, they reduce their walking cadence (− 8.2%) and exhibit longer step length and longer step time than those with DIS ischemia (+ 5.4% and + 5.8% respectively). These latter patients were walking at the classical cadence of healthy old people^14^. Changes in walking cadence affect the frequency of limb progression and ground contacts leading to a reduction in muscular solicitation. A previous study showed that PAD patients present a more prevalent unsteadiness and history of fallings^15^. As such, we can assume that a reduced gait cadence in patients with PROXDIS ischemia results from a behavioral adaptation to produce a more stabilizing gait pattern in order to reduce ischemic pain sensation and its development. Such a compensatory phenomenon is observed in other parameters. When studying diabetic patients with peripheral neuropathy and a forefoot ulcer, Hastings et al. showed significantly increased FPA compared to controls (15(9)° vs 9(4)°)^16^. They hypothesized that this increase in FPA results from a diminished plantar flexion motion to preserve gait stability. We observed FPA in patients with PROXDIS ischemia in the same range as in the aforementioned control population. Many factors could impact FPA values (such as joint axis deformity, tibia and/or femoral torsion, mid-foot deformity, etc.). Nevertheless, the fact that FPA was significantly higher in cases of DIS ischemia (11.8(6.8)° in DIS vs 8.8(4.4)° in PROXDIS) suggests that buttocks-located ischemia affects this compensatory mechanism.

The location of ischemia also impacts sEMG parameters. IC patients with PROXDIS ischemia present a decreased TA activation peak and time (− 4.5% and − 19.7%) compared to DIS ischemia. Indeed, as we previously discussed, these patients adopt a specific gait pattern to reduce muscular solicitation. In the PROXDIS group, ischemia and pain are distributed in two sites in the leg, leading to more extended and global muscular weakness than in the DIS group. This likely explains the lower and shorter neuromuscular activation observed at the TA level and the greater gait alterations in the PROXDIS group.

The location of ischemia corresponds strongly to the location of pain described by patients. To our knowledge, no study has investigated the relationship between changes in neuromuscular activation in ischemic lower limbs and the location of ischemia when pain occurred during walking. Our results suggest that PROXDIS ischemia induces more noxious alterations in neuromuscular performance during walking than DIS ischemia, as confirmed by a shorter maximum walking distance (-40.1%), which is an indicator of disease progression and of exercise capacity^17^. When focusing on the influence of pain on neuromuscular activity, previous studies have indicated that muscle activity is not reorganized in a simple systematic redistribution from painful to non-painful muscles and that differences between muscle adaptations to pain might depend on neurophysiological constraints^18,19^. It appears that ischemia can be considered as such a neurophysiological constraint and different ischemia locations might explain the different neuromuscular adaptation pattern between groups in our study.

During the treadmill-walking test, patients suffer ischemia-induced pain and this pain will increase with exercise prolongation (and thus muscular solicitation) until a threshold leading the patients to stop the test. Such a time-effect related to ischemic pain development is likely to affect gait parameters but it has never been investigated before. We observed that the onset of pain did not impact gait parameters directly. Some authors have previously shown that pain during walking affects gait parameters such as step length and cadence^12,13^, while others^8,20^ found no difference between the pain-free phase and the pain phase. In our study, when analyzing ground reaction forces, a decrease occurred for the two peak forces (corresponding to braking and propulsive forces) with the increase in ischemic pain intensity until maximum pain. These results corroborate the decrease in the second peak force during the pain phase compared to the pain-free phase in IC patients^7^. Similarly, it has been demonstrated that the ischemic limb exhibited a decrease in propulsive forces in the forefoot during gait, but without any correlation with the onset of claudication pain^6^.

As revealed by sEMG activity, onset of pain did not affect neuromuscular activation; however, the increasing ischemic pain, with prolongation of exercise, induced modifications in TA and GM activation. When walking, TA ensures support during the early stance (corresponding to the first peak force) and GM plays an important role in propulsion (second peak force)^21,22^. Between the pain-free and maximum pain phases, sEMG activation amplitude was reduced in TA in patients with PROXDIS ischemia only (− 13.0%) as well as sEMG activation peak and time in GM in both groups (− 3.5% and − 12.3% respectively). Despite the fact that these results fit well with the reduction of plantar peak forces reported, and with lower activation capacity in IC patients^10^, this will warrant further investigations since on the opposite it has been shown that TA activity duration decreased with IC, and sEMG amplitudes increased for TA and GM muscles^11^.

Many theories about motor adaptations to pain suggest that these adaptations present short-term benefits (reduction of pain and load on painful muscles). However, they can also induce negative long-term consequences such as an increase in the biomechanical constraint at tendon and muscle levels in non-painful areas and then a higher risk of injuries^23^. Knowing this, defining and characterizing neuromuscular adaptations to ischemic pain in IC patients may allow a better understanding of the motor strategies used to compensate and reduce pain feel in the lower limbs.

Finally, rehabilitation through exercise therapy is well recognized as an effective method to improve gait in IC^24,25^. Indeed, it has been shown that IC patients can benefit from physical activity programs at various levels: it increased maximum calf muscle blood flow, oxygen extraction levels^26^, claudication onset distance and total walking distance^27^, strength, endurance, and coordination of lower-limb muscles^28^. The results of our study indicate that DIS and PROXDIS ischemia affects walking differently in patients with IC. However, these programs never take into account the location of ischemia and the intensity of ischemic pain as adaptation parameters. Setting up a physical rehabilitation program in patients with IC on the basis of these parameters would therefore be interesting in order to test its effectiveness compared to current protocols and to improve care.

In our study, we include ten patients with DIS ischemia and 12 with PROXDIS ischemia. This rather small number of patients in our analysis could be perceived as a limitation. On the contrary, we assume that this reveals the quality of our patient’s selection and this is an asset of major importance. Indeed, in most of the studies analyzing gait parameters in PAD-IC, patients were often recruited only on their ABI value and associated symptoms^5,8,29^. However, we know that:

- Exercise ischemia can occur even in absence of pathological ABI in the asymptomatic leg (non-painful) in a significant proportion (46.7%)^30^ and then ischemia that is clinically considered unilateral could be bilateral.
- In proximal ischemia, ABI is frequently non-pathological and iliac arteries could be difficult to visualize, therefore, a great deal of proximal ischemia is not detected^31,32^.
- Some studies referred to symptomatic and asymptomatic legs, which is different from ischemic or non-ischemic legs AND we believe that the proof of underlying ischemia as the cause of pain (as reported here) is of major importance in elderly patients when so many co-morbid (mostly osteoarticular) conditions may be painful with exercise.
- Some studies find that gait parameters^12,33^ were affected differently by claudication pain between symptomatic and asymptomatic legs while others were not^8,13,34^, probably simply because the presence and location of exercise ischemia could not be determined.

To avoid any risk of false ischemia classification, we recorded transcutaneous pressure in dioxygen in both limbs all along the procedure to assess the occurrence of ischemia and we studied gait alterations based on this. In addition, concordance between patients’ description of pain (due to ischemia) during walking and the location of ischemia was checked. Consequently, this high level of selectivity leads us to present relatively small populations but with the advantage of being highly specific.

Patients with IC are frequently more than 55 years old ([52–80] years old in our study). Elderly people present gait alterations, similar to those of PAD patients^14,35^. In addition, age-related changes in neuromuscular activation can also be present during walking^35^. However, a recent study found that PAD impacts gait parameters more than age^36^ and our results are consistent with the current literature on PAD. Consequently, we assume that our results stem mainly from PAD and not age.

Diabetes could also possibly affect our results. Because of similar risk factors (age, smoking, overweight BMI, etc.), PAD patients are frequently affected by diabetes. Nevertheless, even if diabetes, with or without neuropathy, can induce gait alterations^37^, there was no change in gait parameters in PAD patients when they were adjusted for diabetes^38^. Based upon these observations, we deliberately chose not to exclude patients with diabetes.

Patients with IC present gait parameters and neuromuscular alterations in ischemic lower limbs during walking. Our main finding is that these alterations, leading to specific neuromuscular adaptations, are different according to the location of ischemia and ischemic pain intensity. PROXDIS ischemia leads to lower walking cadence, longer step length and step time, shorter maximum walking distance and decreased TA activation peak and time than DIS ischemia. In addition, the fact that FPA was significantly higher in cases of DIS ischemia suggests that buttocks-located ischemia affects this compensatory mechanism. Therefore, in patients with PROXDIS ischemia, gait parameters and muscular activation are more dramatically impacted than in patients with DIS ischemia. Additionally, while the onset of ischemic pain does not affect gait, its increase, with prolongation of exercise, leads to alterations (reduced peak forces in both groups, reduced sEMG activation amplitude in TA in PROXDIS ischemia only and decreased sEMG activation peak and time in GM in both groups).

Finally, these findings could then be used to adapt rehabilitation programs and improve their specificity and efficiency.

## Data Availability

The datasets generated and/or analyzed during the current study are available from the corresponding author on reasonable request.
